# Atbf1 Regulates Pubertal Mammary Gland Development Likely by Inhibiting the Pro-Proliferative Function of Estrogen-ER Signaling

**DOI:** 10.1371/journal.pone.0051283

**Published:** 2012-12-12

**Authors:** Mei Li, Xiaoying Fu, Gui Ma, Xiaodong Sun, Xueyuan Dong, Tamas Nagy, Changsheng Xing, Jie Li, Jin-Tang Dong

**Affiliations:** 1 Department of Genetics and Cell Biology, College of Life Sciences, Nankai University, Tianjin, China; 2 Department of Hematology and Medical Oncology, Emory Winship Cancer Institute, Emory University School of Medicine, Atlanta, Georgia, United States of America; 3 Department of Pathology, College of Veterinary Medicine, University of Georgia, Athens, Georgia, United States of America; The University of Hong Kong, Hong Kong

## Abstract

ATBF1 is a candidate tumor suppressor that interacts with estrogen receptor (ER) to inhibit the function of estrogen-ER signaling in gene regulation and cell proliferation control in human breast cancer cells. We therefore tested whether Atbf1 and its interaction with ER modulate the development of pubertal mammary gland, where estrogen is the predominant steroid hormone. In an *in vitro* model of cell differentiation, i.e., MCF10A cells cultured in Matrigel, *ATBF1* expression was significantly increased, and knockdown of *ATBF1* inhibited acinus formation. During mouse mammary gland development, *Atbf1* was expressed at varying levels at different stages, with higher levels during puberty, lower during pregnancy, and the highest during lactation. Knockout of *Atbf1* at the onset of puberty enhanced ductal elongation and bifurcation and promoted cell proliferation in both ducts and terminal end buds of pubertal mammary glands. Enhanced cell proliferation primarily occurred in ER-positive cells and was accompanied by increased expression of ER target genes. Furthermore, inactivation of *Atbf1* reduced the expression of basal cell markers (CK5, CK14 and CD44) but not luminal cell markers. These findings indicate that Atbf1 plays a role in the development of pubertal mammary gland likely by modulating the function of estrogen-ER signaling in luminal cells and by modulating gene expression in basal cells.

## Introduction

AT-motif binding factor 1 (ATBF1), also named ZFHX3 for zinc finger homeobox 3, was originally identified as a transcriptional repressor of the human alpha-fetoprotein (*AFP*) gene [1], [2]. It encodes a protein of 3703 amino acids comprising a large number of structural domains such as zinc fingers and homeodomains. The *ATBF1* gene was later suggested to be a strong candidate tumor suppressor gene in human cancers because it is frequently mutated in prostate cancer, and its chromosomal locus is frequently deleted and its expression significantly downregulated in multiple types of tumors [3], [4], [5], [6], [7]. Functionally, ATBF1 cooperates with p53 to activate the p21^Waf1/Cip1^ CDK inhibitor to arrest the cell cycle [8], [9] and inhibits the signal transducer and activator of transcription 3 (STAT3) signaling by interacting with PIAS3 (protein inhibitor of activated STAT 3) [10]. ATBF1 can also modulate cell differentiation and is induced in neuronal differentiation [11], [12], [13], [14]; it regulates aminopeptidase N (APN), a marker of enterocyte differentiation and maturation in the small intestine [15]; it affects pituitary gland differentiation by regulating the pituitary lineage determining factor 1 (Pit1) [16]; and its knockout in mouse prostates dysregulates a number of differentiation genes (Sun et al., unpublished data).

In human breast cancer, although *ATBF1* is infrequently mutated [4], its genomic locus is deleted in as high as 75% of ductal cancers and 100% of lobular cancers [6], [17]. In addition, *ATBF1* mRNA expression is often downregulated in human breast cancer, and the downregulation is associated with adverse features of breast cancer such as higher tumor stage and grade, larger tumor volumes, metastasis, and worse patient survival [7]. Interestingly, higher levels of ATBF1 expression were associated with estrogen receptor alpha (ERα, hereafter ER) positivity in breast cancer [7], and ATBF1 and the estrogen-ER signaling appear to form an autoregulatory feedback loop relationship [18], [19], [20]. On one hand, ATBF1 interacts with ER to inhibit the function of estrogen-ER signaling in gene regulation and cell proliferation control [20]. Proper ER function also appears to require fine-tuned levels of ATBF1, because ER induces *ATBF1* transcription but causes ATBF1 protein degradation via the proteasome by inducing the estrogen responsive finger protein (EFP) [18], [19].

Postnatal mammary gland development involves a number of different stages such as ductal elongation and bifurcation during puberty, side branching during estrous cycles, and alveologenesis and lactogenesis during pregnancy and lactation [21]. It is highly regulated by reproductive steroids including estrogen, progesterone (Pg) and prolactin (PRL) through their receptors ER, PR and PrlR respectively. Hormonal signaling activates different factors to induce proliferation in some cells and differentiation in other cells, and a number of factors have been discovered for different functions of hormonal signaling, including GATA binding protein 3 (Gata3) (necessary in both virgin and pregnant mice), signal transducer and activator of transcription 5a/b (Stat5a/b) and E74-like factor 5 (Elf5) (modulating alveolar development during pregnancy) [22]. Different hormones have different impacts on different stages of mammary gland development [23], [24]. Estrogen-ER signaling has been shown to play a more dominant role during puberty [21]. Taken together with the fact that ATBF1 is dysregulated in breast cancer and that ATBF1 and ER have an autoregulatory feedback loop, we hypothesize ATBF1 plays a role in mammary gland development during puberty.

In this study, we evaluated *Atbf1* expression in mammary glands and examined the role of Atbf1 in the development of pubertal mammary gland by using *in vitro* and *in vivo* models. We found that *Atbf1* expression varied during cell differentiation and mammary gland development. Furthermore, deletion of *Atbf1* in mouse mammary gland promoted ductal elongation/bifurcation, likely by enhancing the pro-proliferative function of estrogen-ER signaling, and attenuated the expression of basal cell markers in pubertal mammary gland. These findings indicate a regulatory role for Atbf1 in mammary gland development at least during the puberty.

## Materials and Methods

### Ethics statement

Mice used in these studies were housed at the Division of Animal Resources (DAR) facility at Emory University and handled by DAR stuff. All mice were closely monitored and humanely euthanized. All experimental procedures involving animals were approved by the Institutional Animal Care and Use Committee (IACUC No. 2001337).

### Reagents, antibodies, cell lines and mice

The following reagents were purchased from their respective vendors: Matrigel with reduced growth factor (BD Biosciences, San Jose, CA); Cell counting kit-8 (Dojindo Molecular Technologies, Gaithersburg, MD); Hematoxylin and permount mounting solution (Fisher Scientific, Waltham, MA); Immunohistochemical (IHC) staining reagents (Dako, Carpinteria, CA); Alexa Fluor Dye (Invitrogen, Carlsbad, CA); Fluoro-gel with TES buffer (Electron Microscopy Sciences, Hatfield, PA); β-casein antibody (Santa Cruz, Santa Cruz, CA); Ki67 antibody (Fisher Scientific); ERα antibody (Santa Cruz); CK5 antibody (Covance Research Products, Princeton, NJ); Human CK18 antibody (Dako); Mouse CK18 antibody (GeneTex, Irvine, CA). Generation of ATBF1 antibody, negative siRNA and ATBF1 siRNA was previously described [20].

The immortalized but non-tumorigenic human breast epithelial cell line MCF10A was purchased from ATCC (Manassas, VA) and cultured in DMEM/F12 medium supplemented with 5% horse serum, 20 ng/ml epidermal growth factor (EGF), 10 µg/ml insulin, 0.5 µg/ml hydrocortisone, and 100 ng/ml cholera toxin as described previously [25]. B6/129-Tg(MMTV-cre)4Mam/J (MMTV-Cre hereafter) mice, which specifically express the Cre in mammary epithelial cells, were purchased from the Jackson Lab (Bar Harbor, ME).

### Culture of MCF10A cells in Matrigel (3-D culture)

Briefly, 40 µl of Matrigel with reduced growth factor were added to each well of eight-well glass chamber slides and spread evenly. After Matrigel was solidified for 15 min, 5000 cells in assay medium containing 5 ng/ml EGF and 2% Matrigel were seeded in each well, and the medium was replaced every three days. Images of spheres with defined scales were subjected to the ImageJ computer program to determine the area covered by each sphere, and the diameter of that sphere was then calculated based on the circle formula.

### Mouse breeding and genotyping

Generation of mice containing the floxed *Atbf1* allele (*Atbf1^f^*) was previously described [26]. Floxed *Atbf1* mice that had been crossed back to the C57BL/6J background were crossed with MMTV-*Cre^+^* mice, and the F1 mice were then intercrossed. Female mice with the following genotypes, *Cre^+^/Atbf1^wt/wt^* (defined as *Atbf1* ^+/+^, wild-type), *Cre^+^/Atbf1^wt/f^* (defined as *Atbf1* ^+/−^, heterozygous deletion), and *Cre^+^/Atbf1^f/f^* (defined as *Atbf1* ^−/−^, homozygous deletion), were collected. Mice were genotyped by PCR with tail DNA and mammary gland genomic DNA using the following primers: 5′-GGCCCTTGACTGCATTTCTTTCCTGT-3′ and 5′-ATTCGTTAATGGGAAGGTGTCAGA-3′ (*Atbf1*); 5′-CGGTCGATGCAACGAGTGAT-3′ and 5′-CCACCGTCAGTACGT GAGAT-3′ (*Cre*); and 5′-CTAGGCCACAGAATTGAAAGATCT-3′ and 5′-GTAGGTGGAAATTCTAGCATCATCC-3′ (*Il-2*, as the internal control).

### Whole mount analysis of mammary glands

The fourth abdominal mammary gland on the left was harvested from pubertal mice, placed between two glass slides, and spread by placing weights on top of the slides for 10 min, followed by fixing in 3.7% formalin overnight. Fixed tissues were washed in serially diluted ethanol and stained at room temperature with hematoxylin for 5–10 min. Stained tissues were washed by flowing water, dehydrated progressively in graded alcohol solutions (70%, 95% and 100%; 30 min each), and cleared in xylene for at least 30 min before mounting with cover slips. Slides with whole mount mammary glands were scanned at a 2400 dpi resolution.

### Immunohistochemical (IHC) and Immunofluorescence (IF) staining

Tissue sections were prepared from formalin-fixed paraffin-embedded tissues for IHC and IF staining. Briefly, tissue sections were first deparaffinized and rehydrated following the standard procedure. After the treatment with blocking buffer, sections were heated in sodium citrate buffer for antigen retrieval. For IHC staining, sections were incubated with ATBF1 antibody (1∶1000) at 4°C overnight and then with the HRP solution, DAB-chromogen, hematoxylin, dehydrated and finally stored in Permount mounting solution. For IF, the blocking solution was 10% normal goat serum in PBS and the secondary antibody was conjugated with the Alexa Fluor Dye. Nuclei were stained with diamidinophenyl-indole (DAPI) and sections in the anti-fade mounting solution were stored at −20°C in the dark.

### RNA extraction, RT-PCR and Real time RT-PCR

The fourth abdominal mammary gland on the right, without lymph node, was harvested for RNA and protein analysis. For RNA extraction, harvested tissues were immediately submerged in RNAlater (RNA stabilization reagent). Total RNA from the mammary glands was extracted using the RNeasy Mini Kit following the manufacturer's instructions (Qiagen, Valencia, CA). The first strand cDNA was synthesized by using the iScript cDNA synthesis kit (Bio-Rad, Richmond, CA). Primer sequences for semi-quantitative PCR to determine the deletion of *Atbf1* at the RNA levels were 5′-GGCCAGATCTTCACCATCC-3′ (forward) and 5′-CAGGGAGGAACATGCTACTAGG-3′ (reverse). Primer sequences for real time RT-PCR are shown in Table 1.

**Table 1:** Primer sequences for real time RT-PCR.

Gene	Forward	Reverse
**hATBF1**	TGTTCCAGATCGAGATGGGAAT	CTTTCCCAGATCCTCTGAGGTTT
**hCK18**	CGCCAGGCCCAGGAGTATGAGG	ACTATCCGGCGGGTGGTGGTCTTT
**hCD44**	CAACTCCATCTGTGCAGCAAA	GTAACCTCCTGAAGTGCTGCTC
**hCK14**	TTCTGAACGAGATGCGTGAC	GCAGCTCAATCTCCAGGTTC
**hCK5**	TGGTCTCCCGTGCCGCAGTTCTAT	ATTTGGGATTGGGGTGGGGATTCT
**hCD24**	TGAAGAACATGTGAGAGGTTTGAC	GAAAACTGAATCTCCATTCCACAA
**hCK18**	CGCCAGGCCCAGGAGTATGAGG	ACTATCCGGCGGGTGGTGGTCTTT
**hCK8**	GCTGACCGACGAGATCAACT	CATGGACAGCACCACAGATG
**hGAPDH**	GGTGGTCTCCTCTGACTTCAACA	GTTGCTGTAGCCAAATTCGTTGT
**mAtbf1 (regular)**	GGCCAGATCTTCACCATCC	CAGGGAGGAACATGCTACTAGG
**mAtbf1 (real time)**	AGAGCAAGAGGGCAGCGTCATC	CGGTTCACGTCAGCGTTGCTATAC
**mER**	TTCTGTCCAGCACCTTGAAGTCTCTG	CATCTCCAGGAGCAGGTCATAGAGG
**mCK5**	GGAGCTGGCTCTCAAAGATG	TCCAGCAGCTTCCTGTAGGT
**mCK14**	GCTCTTGTGGTATCGGTGGT	GAGGAGAAGCGAGAGGAGGT
**mCD44**	TGGATCCGAATTAGCTGGAC	AGCTTTTTCTTCTGCCCACA
**mCK8**	ATCGAGATCACCACCTACCG	TGAAGCCAGGGCTAGTGAGT
**mCK18**	CGAGGCACTCAAGGAAGAAC	CTTGGTGGTGACAACTGTGG
**mCD24**	GCAACCACAAGTCCAATGTG	TTTCACGCGTCCTTTAATCC
**mAreg**	GACTCACAGCGAGGATGACA	GGCTTGGCAATGATTCAACT
**mEbag9**	GAGTGGACTTCCTGGGATGA	AAAACCCGTGCTACCATCTG
**mIgf-1**	TCATGTCGTCTTCACACCTCTTCT	CCACACACGAACTGAAGAGCAT
**mMyc**	GCCCAGTGAGGATATCTGGA	ATCGCAGATGAAGCTCTGGT
**mGapdh**	CCAGTATGACTCCACTCACG	GACTCCACGACATACTCAGC

### Protein extraction and western blotting

Western blotting was performed as previously described [27], [28]. Briefly, harvested tissues were flash-frozen in liquid nitrogen and lysed in cold RIPA buffer (20 mM Tris pH 8.0, 150 mM NaCl, 0.5% sodium deoxyocholate, 0.1% SDS, 1% NP-40, 10 mM sodium pyrophosphate, and 10 mM sodium fluoride) supplemented with protease/phosphatase inhibitors. Cultured cells were washed with PBS and lysed in the same RIPA buffer. Lysates were incubated on ice for 20 minutes with frequent vortexing and cleared by centrifugation. Proteins were separated by 4% SDS-PAGE for ATBF1 and 10% SDS-PAGE for β-actin. The ATBF1 antibody was at 1∶800 dilution in 3% BSA/PBS and β-actin antibody was at 1∶10,000.

### Statistical analysis

Statistical analyses were performed using the SPSS® statistical software (SPSS Inc., Chicago, IL, USA). Student's *t* test was used to determine statistical differences between two groups, whereas one-way ANOVA or univariate analysis was used to compare three or more groups, as detailed in figure legends. *P* values less than 0.05 were considered statistically significant.

## Results

### Induction of ATBF1 expression during MCF10A cell differentiation

**Figure 1:**
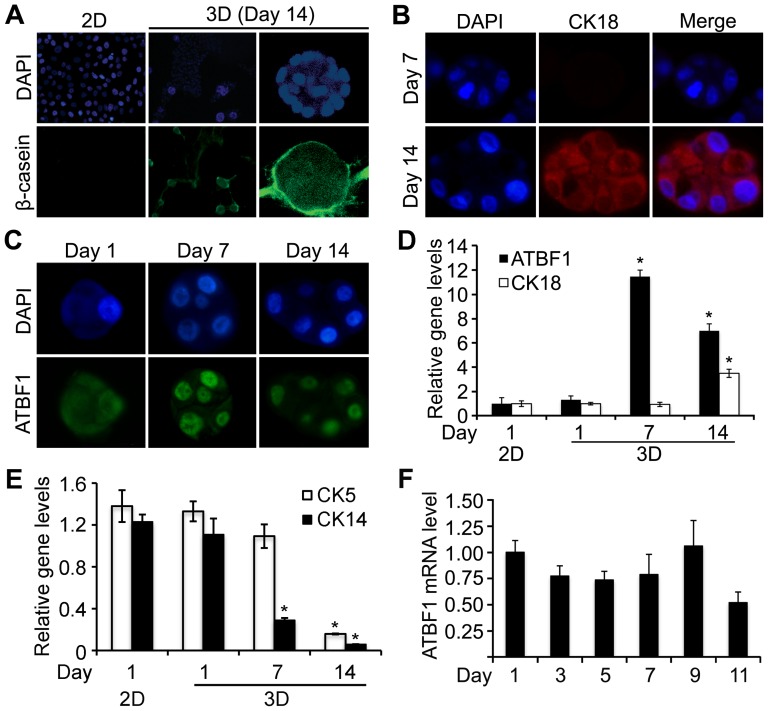
ATBF1 is significantly induced during the differentiation of MCF10A cells in a 3-D culture model. (A–C) Immunofluorescence staining was used to detect the expression of differentiation markers milk protein β-casein (lower row) (green) (A) and CK18 (red) (B) as well as ATBF1 (green) (C). DAPI staining (blue) shows nuclei of cells. (D–F) Relative mRNA expression of *ATBF1*, *CK18*, *CK5*, and *CK14* in MCF10A cells from 2-D and 3-D cultures of indicated times (D and E) or 2-D culture of different times (F), as detected by real time RT-PCR. *GAPDH* was used as an internal control. Error bar represents SEM. * indicates *P*<0.01 (student's *t* test).

MCF10A cells cultured in Matrigel (3-D) proliferate and differentiate to form spheres with acini, and thus have been used as an *in vitro* model that mimics mammary epithelial differentiation [25], [29], [30]. To test whether ATBF1 plays a role in mammary epithelial differentiation, we first examined the expression of ATBF1 in MCF10A cells in both 2-D (plastic plate) and 3-D (Matrigel) cultures. In the 3-D culture, differentiation of MCF10A cells was indicated by the formation of acini (Fig. 1A–1C) and the induction of differentiation markers β-casein in the space surrounding acini (Fig. 1A) and CK18 in the cytoplasm (Fig. 1B, 1D). Induction of β-casein and CK18 was detectable at day 14 but not at day 7. Expression of basal markers *CK14* and *CK5*, on the other hand, was decreased during the differentiation (Fig. 1E). For ATBF1, a significant induction was detected at day 7, and the induction was still demonstrable at day 14, although at a lower level (Fig. 1C, 1D). In the 2-D culture, *ATBF1* expression was detected at a lower level (Fig. 1D) without an increase during a 9-day period of culture (Fig. 1F), during which time cells showed a typical proliferation curve as expected (data not shown). These results suggest that the induction of ATBF1 is related to cell differentiation.

**Figure 2:**
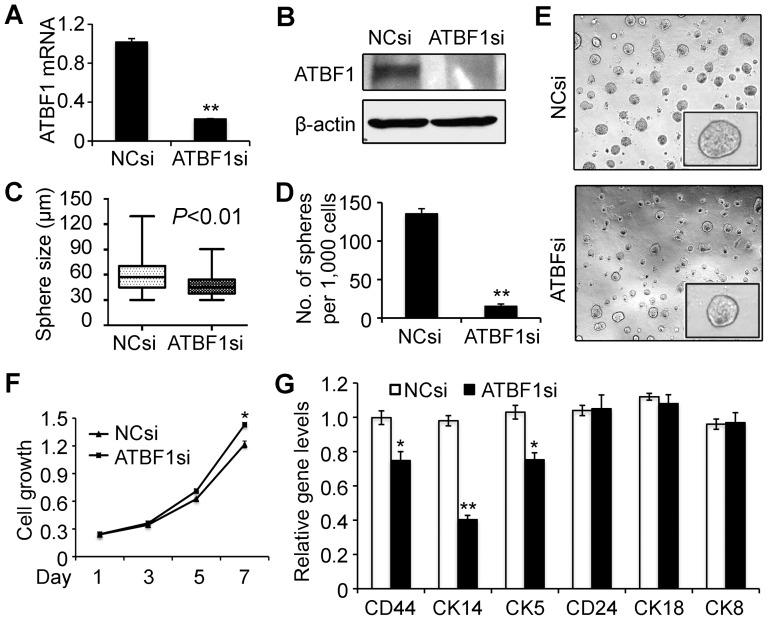
Knockdown of *ATBF1* interrupts mammosphere formation and reduces the expression of basal cell markers. (A, B) Cells transfected with negative control siRNA (NCsi) or *ATBF1* siRNA (ATBF1si) were subjected to real time RT-PCR for mRNA expression (A) and western blotting for protein expression (B). (C) Distribution of diameters of all mammospheres at 2-weeks of culture in Matrigel. The average sphere size for *ATBF1* knockdown is significantly smaller than that for the control. (D) Knockdown of *ATBF1* significantly reduces the number of spheres with a diameter >75 µm. The data are presented as the number of defined mammospheres per 1,000 seeded cells ± SEM. (E) Representative bright field images for spheres from both groups. (F) Measurement of cell numbers for MCF10A cells treated with NCsi or ATBF1si in 24-well plates (2-D culture). (G) Expression of both basal cell markers (*CD44*, *CK14* and *CK5*) and luminal cell markers (*CD24, CK18 and CK8*) in MCF10A cells in Matrigel, as detected by real time RT-PCR. * and ** indicate *P*<0.05 and *P*<0.01 respectively (student's *t* test).

To determine the role of induced ATBF1 in the differentiation of MCF10A cells in Matrigel, we knocked down *ATBF1* by transfecting siRNAs into MCF10A cells before they were plated into Matrigel (Fig. 2A, 2B). Interference with *ATBF1* expression resulted in a significant inhibition of mammosphere formation of MCF10A cells in Matrigel, as indicated by the number of spheres with a diameter greater than 75 µm (Fig. 2C–2E). In the 2-D culture, however, MCF10A cell proliferation was slightly enhanced by the knockdown of *ATBF1* (Fig. 2F) suggesting that the attenuation of mammosphere formation by *ATBF1* knockdown may not be caused by the increase in cell proliferation but likely by a change in cell differentiation. Furthermore, knockdown of *ATBF1* reduced the expression of basal cell markers *CD44*, *CK14* and *CK5*, but not that of luminal cell markers *CD24*, *CK18* or *CK8* (Fig. 2G). These results suggest that ATBF1 plays a role in the differentiation of mammary epithelial cells, which might involve the maintenance of the basal cell layer.

### Expression of *Atbf1* in mouse mammary glands

**Figure 3:**
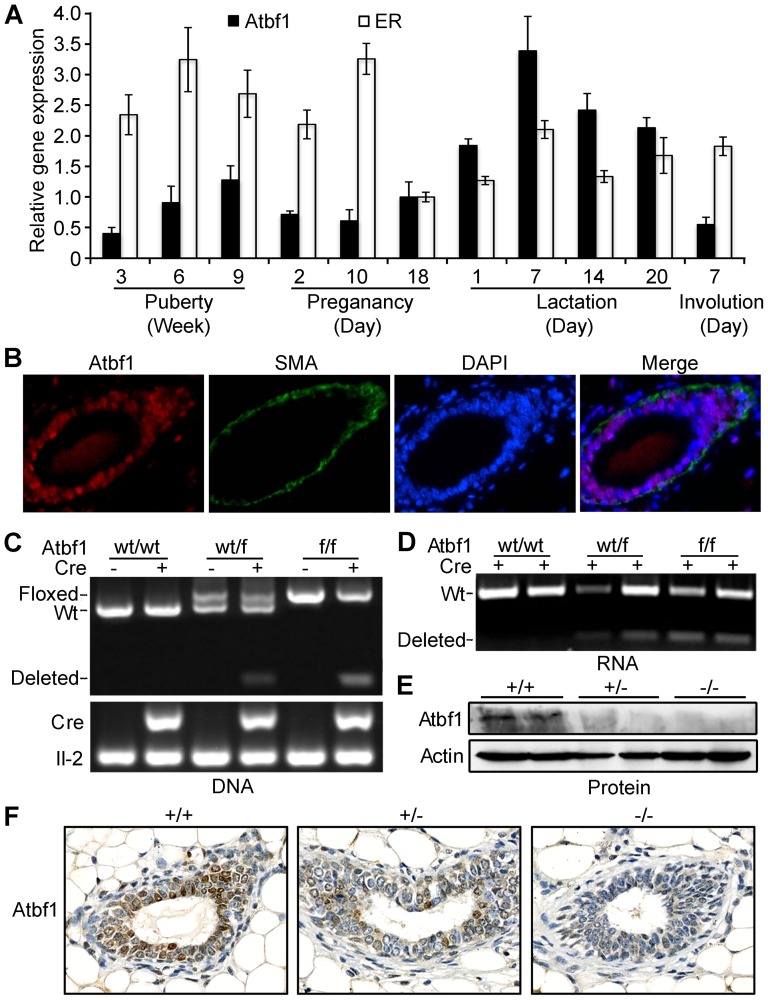
Validation of mammary epithelial cell-specific deletion of *Atbf1* in mice. (A) Increased expression of *Atbf1* mRNA in mammary glands of C57BL/6J female mice during puberty and the first week of lactation, as measured by real time RT-PCR. Three mice were used for each time point. Expression of *ER*, which changes at different stages, was used as a positive control. *P* values for comparisons between puberty and lactation, between pregnancy and lactation, and between puberty and pregnancy are <0.005, <0.005, and 0.962, respectively. (B) Double IF staining of Atbf1 (red) and SMA (green) shows the expression and localization of Atbf1 in mouse mammary gland. (C) Deletion of *Atbf1* genomic DNA upon the expression of Cre in mammary tissues, as detected by PCR. The upper panel shows the wild-type *Atbf1* allele (Wt), floxed allele without deletion (Floxed), and the allele with Cre-mediated deletion (Deleted). The lower panel shows the presence of the *Cre* gene in a mouse, with the interleukin-2 gene (*Il-2*) as a PCR control. Genotypes and *Cre* status are indicated at the top. (D) Detection of truncated *Atbf1* mRNA in mouse tissues expressing the *Cre* gene under the MMTV promoter. All mice are positive for *MMTV-Cre*. “+” and “−” indicate wild-type (Wt) and deleted *Atbf1* mRNA respectively. Each lane represents one mouse and two mice were used for each genotype. (E, F) Reduced Atbf1 protein expression by Cre-mediated deletion, as detected by western blotting (E) and immunohistochemical staining (F) with antibody against ATBF1. Thoracic mammary glands from mice with wild-type (+/+), heterozygous (+/−) and homozygous (−/−) *Atbf1* were used in these analyses.

In order to test the function of Atbf1 in a mouse model, we first evaluated the expression of Atbf1 during mammary gland development in mouse. We collected mammary glands at four different stages (virgin, pregnancy, lactation and involution), and performed real time RT-PCR to measure *Atbf1* mRNA expression. The expression of *ER* was used as a control for gene expression in mammary tissues at different stages. As expected, *ER* expression was higher during puberty and pregnancy but lower during lactation (Fig. 3A). The level of *Atbf1* expression also varied at different stages, with an increase from week 3 to week 9 during puberty but relatively lower levels during pregnancy. During lactation, *Atbf1* mRNA was at significantly higher levels, with the highest at day 7 (Fig. 3A). Moreover, IF staining showed that Atbf1 was expressed in the nucleus of both luminal and myoepithelial cells, with strong staining in some luminal cells (Fig. 3B). Dynamic *Atbf1* expression during mammary gland development suggests that Atbf1 plays different roles in different stages of developing mammary gland, and is likely more relevant to puberty and lactation.

### Conditional knockout of *Atbf1* in mouse mammary glands

To better understand the function of Atbf1 in mammary gland development, we bred floxed *Atbf1* mice [26] to *MMTV-Cre* mice to specifically knock out *Atbf1* in mouse mammary epithelial cells. To detect Cre-mediated *Atbf1* deletion, we designed a pair of PCR primers, of which one was upstream to the first *loxP* site while the other was downstream to the second *loxP* site. The primers could produce a small PCR product (289 bp) when the floxed *Atbf1* allele was deleted by Cre. As expected, breeding with *Cre* mice resulted in PCR products indicative of *Atbf1*'s genomic deletion (Fig. 3C) and truncated *Atbf1* mRNA (Fig. 3D) in mammary gland tissues with both heterozygous and homozygous *Atbf1* deletion (+/− and −/− respectively). At the protein level, both western blotting and IHC staining revealed that knockout of *Atbf1* significantly reduced Atbf1 protein expression in mammary glands even when the deletion occurred in one of the two alleles (Fig. 3E, 3F).

### Knockout of *Atbf1* promotes ductal elongation and bifurcation in pubertal mammary gland

**Figure 4:**
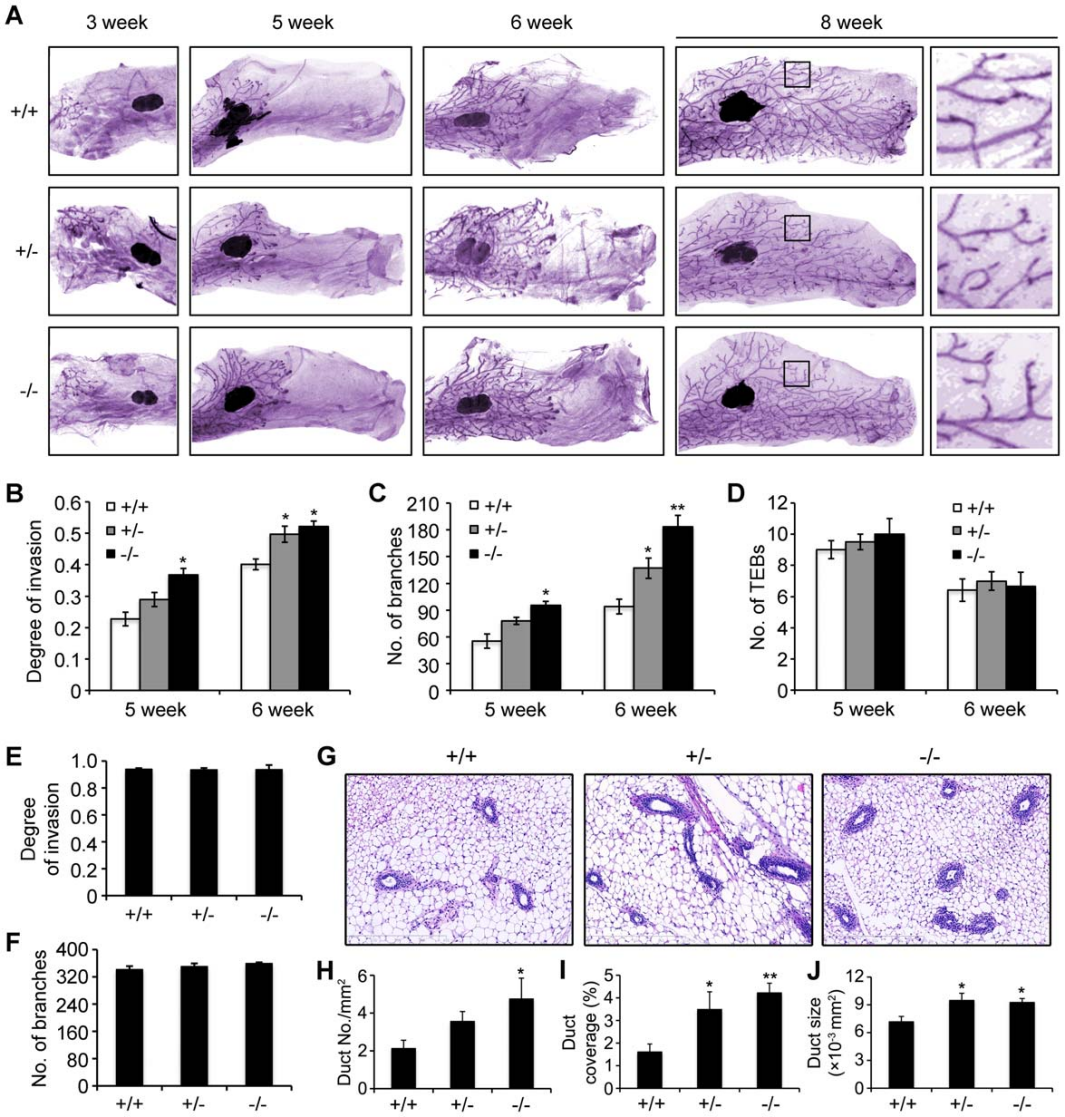
Deletion of *Atbf1* promotes ductal elongation and bifurcation in pubertal mammary gland. (A) Hematoxylin-stained whole-mount images of the fourth abdominal mammary glands from mice at indicated ages. Panels at far right are higher magnification images of indicted areas from 8-week old mammary glands. Three to five mice were used for each genotype. (B–F) Determination of degree of ductal invasion and the numbers of branches and TEBs in mammary glands of mice with indicated genotypes and ages of 5 or 6 weeks (B–D) or 8 weeks (E, F). Degree of ductal invasion was determined by dividing the duct length by the mammary gland length from mid-point of lymph node; and the numbers of total branches and TEBs were determined in whole-mount images by the ImageJ program. (G–J) Comparisons of the numbers and areas of ducts among different genotypes. Shown are representative images from hematoxylin and eosin (HE)-stained tissue sections (Magnification: 100×) from mammary glands at the age of 6-week old, the average number of ducts per mm^2^ (H), ductal area to gland area ratio (ductal coverage) (I), and the average cross-section size per duct (10^−3^ mm^2^) (all ducts were measured for each mouse) (J). HE-stained tissue sections (G) were used for these analyses. Each bar is the average from three mice for each genotype. * and ** indicate *P*<0.05 and *P*<0.01 respectively. For panels H–I, one-way ANOVA was used for statistical analysis, while univariate analysis (sum of squares: type I) was used for panel J.

To investigate the functional contribution of Atbf1 in pubertal mammary gland development, we isolated the fourth abdominal mammary glands from mice at the age of 3, 5, 6 and 8 weeks, performed whole mount analyses, and measured ductal elongation and bifurcation. *Atbf1* deletion significantly accelerated ductal elongation and bifurcation, as shown by direct visualization of ductal trees (Fig. 4A) and statistical analysis of ductal invasion and the number of branches at 5- and 6-week old (Fig. 4B, 4C). *Atbf1* deletion did not affect the number of terminal end buds (TEBs) though (Fig. 4D). At 8 weeks, which marks the end of puberty in mice, ductal extension and branching equalized, and the effect of *Atbf1* deletion disappeared (Fig. 4A, 4E and 4F). Taken together, these findings suggest that, whereas the number of TEBs is not affected, loss of *Atbf1* enhances ductal elongation and bifurcation in the mammary tree during puberty.

In parallel, we fixed thoracic mammary glands at the age of 6 week, embedded in paraffin, and prepared HE-stained tissue sections for duct morphometrics (Fig. 4G). The average number of ducts per square millimeter (mm^2^) in tissue sections was 2.12, 3.56, and 4.75 for wild-type, heterozygous *Atbf1* deletion, and homozygous *Atbf1* deletion, respectively, and the difference between *Atbf1* ^−/−^ mice and *Atbf1* ^+/+^ mice was statistically significant (Fig. 4H). Measurement of duct coverage, as defined by the percentage of tissue areas covered by ductal areas, demonstrated that *Atbf1* deletion significantly increased duct coverage when compared to normal glands (Fig. 4I). Consistent with the number of ducts per mm^2^, the size of ducts was also significantly increased by the deletion of *Atbf1* (Fig. 4J). These findings further indicate that deletion of *Atbf1* promotes ductal elongation and bifurcation in pubertal mammary gland.

### Knockout of *Atbf1* enhances cell proliferation primarily in ER-positive mammary epithelial cells

**Figure 5:**
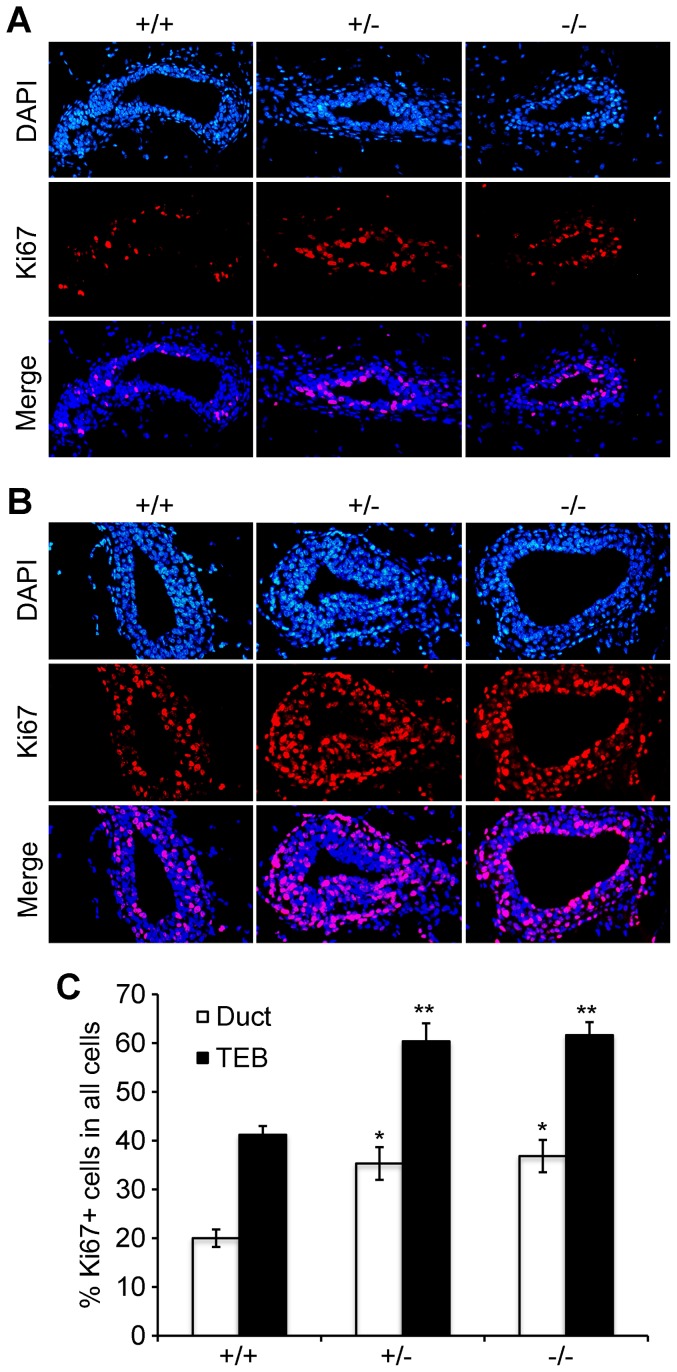
Cell proliferation activity is increased by the knockout of *Atbf1* in mammary epithelial cells from 6-week old mice. (A–B) Tissue sections of mammary ducts (A) and terminal end buds (TEBs) (B) were subjected to IF staining against Ki67. DAPI staining was used to show nuclei (blue) and Ki67 was labeled by RITC (red). (C) Ratios of Ki67-positive cells to total epithelial cells in mammary glands with different *Atbf1* genotypes were calculated. Each bar indicates the average result from 10 randomly selected areas. Three mice were used for each genotype, and the number of mammary epithelial cells counted for each bar was 1200–1600 for ducts and 2000–2800 for TEBs. *, *P*<0.05 and **, *P*<0.01 (one-way ANOVA).

During puberty, the major activities in mammary gland development are ductal elongation and bifurcation accompanied predominantly by cell proliferation. We evaluated cell proliferation in mammary epithelial cells by measuring the Ki67 proliferation marker. In thoracic mammary glands from 6-week old mice, IF staining of tissue sections from nine mice (3 for each genotype) showed that ducts with *Atbf1* knockout (both +/− and −/−) had significantly more Ki67-positive cells than ducts with wild-type *Atbf1* (Fig. 5A, 5C). Similarly, TEBs with *Atbf1* deletion had significantly more Ki67-positive cells than those without deletion (Fig. 5B, 5C).

**Figure 6:**
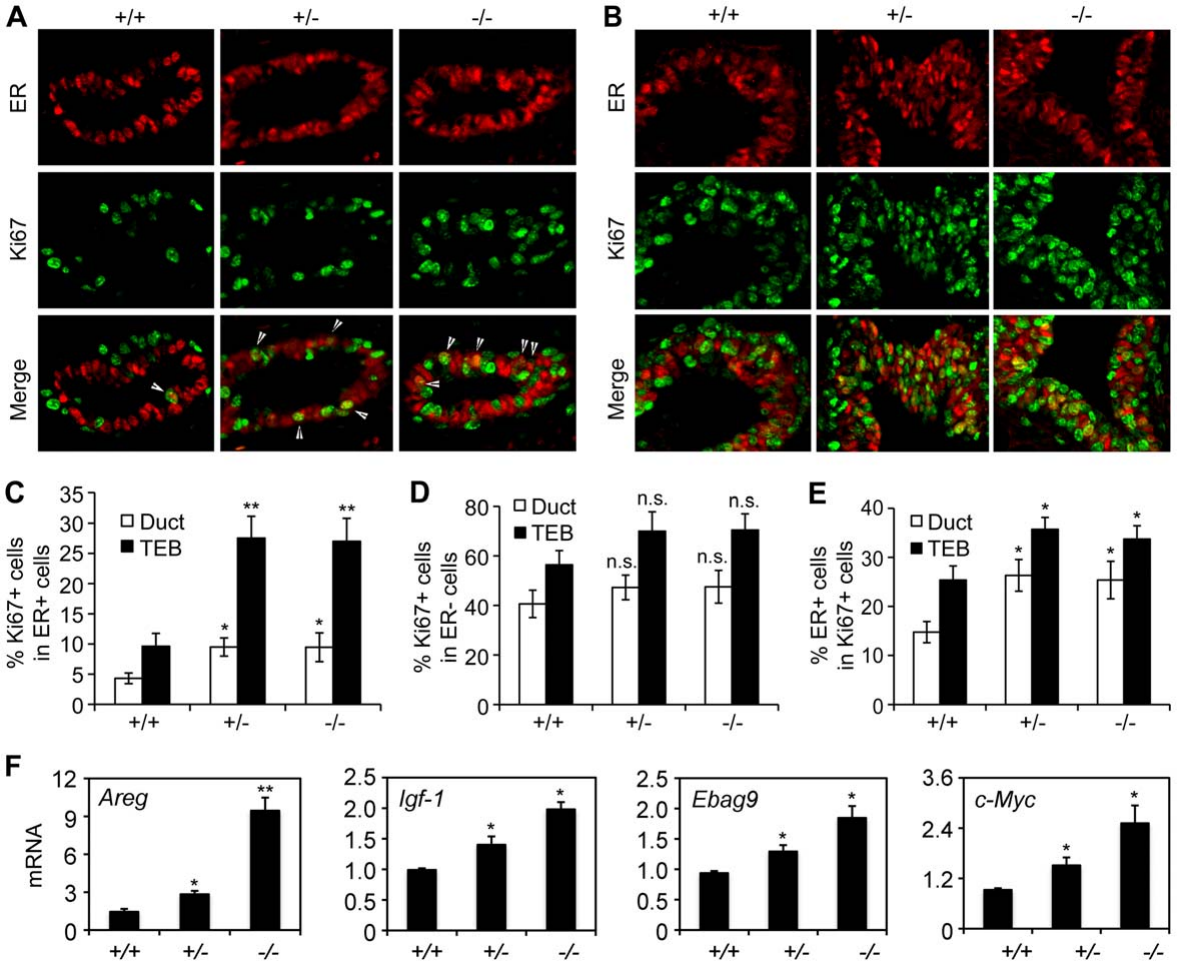
*Atbf1*-knockout-mediated cell proliferation primarily occurs in ER-positive cells. (A, B) Detection of ER (red) and Ki67 (green) by double IF staining in mammary ducts (A) and TEBs (B). White arrows indicate cells that are positive for both ER and Ki67. (C, D) Knockout of *Atbf1* increases cell proliferation in ER-positive cells (C) but not in ER-negative cells (D). (E) Knockout of *Atbf1* also increases the number of ER-positive cells among proliferating (Ki67-positive) cells. For panels C–E, cells were counted from 10 randomly selected fields per mouse, and three mice were used for each genotype. The number of cells counted for each bar ranged from 1000 to 2000 for ducts and 2000 to 2500 for TEBs. *, *P*<0.05; **, *P*<0.01; n.s., *P*>0.05 (one-way ANOVA). (F) Real time RT-PCR was used to detect ER target gene expression (*Areg*, *Igf-1*, *Ebag9* and *c-Myc*).

Estrogen is the most dominant hormone in mammary epithelial proliferation and differentiation during puberty [23]. Our previous *in vitro* studies demonstrated that ATBF1 and estrogen-ER signaling formed an autoregulatory feedback loop to regulate cell proliferation in ER-positive cells [18], [19], [20], and that there was also a correlation between ATBF1 expression and ER positivity in human breast cancer [7]. We therefore evaluated the effect of *Atbf1* knockout on mammary epithelial proliferation in the context of ER activity. We co-stained ER and Ki67 in mammary epithelium cells from ducts and TEBs with wild-type and heterozygous or homozygous *Atbf1* deletion (Fig. 6A, 6B). Statistical analysis showed that the increase in cell proliferation caused by *Atbf1* deletion, as indicated by increased Ki67 positive cells, was only statistically significant in ER-positive cells and not in ER-negative cells, in both ducts and TEBs (Fig. 6C, 6D). As expected, the percentage of ER-positive cells among Ki67-positive cells was significantly higher when *Atbf1* was deleted (Fig. 6E). These results suggest that, during puberty, Atbf1 functions to inhibit cell proliferation primarily in ER-positive mammary epithelial cells.

### Dysregulation of ER target genes and basal cell markers by *Atbf1* deletion in mammary glands

Our previous study indicates that ATBF1 inhibits the function of ER in both cell proliferation and ER target gene expression [20]. Therefore, we evaluated whether knockout of *Atbf1 in vivo* also alters the expression of ER target genes, including amphiregulin (*Areg*), *Igf-1*, *Ebag9*, *Ctsd*, *c-Myc* [20], [31], [32]. *Areg* and *Igf-1* are two well-established estrogen-induced genes that regulate terminal end bud formation and ductal morphogenesis in pubertal mammary gland [33], [34]. Except for *Ctsd*, all other tested ER target genes were significantly up-regulated with the deletion of *Atbf1* (Fig. 6F). *Areg*, in particular, was increased by about 8 fold by homozygous deletion of *Atbf1*. Therefore, Atbf1 also inhibits ER-regulated gene expression *in vivo*.

**Figure 7:**
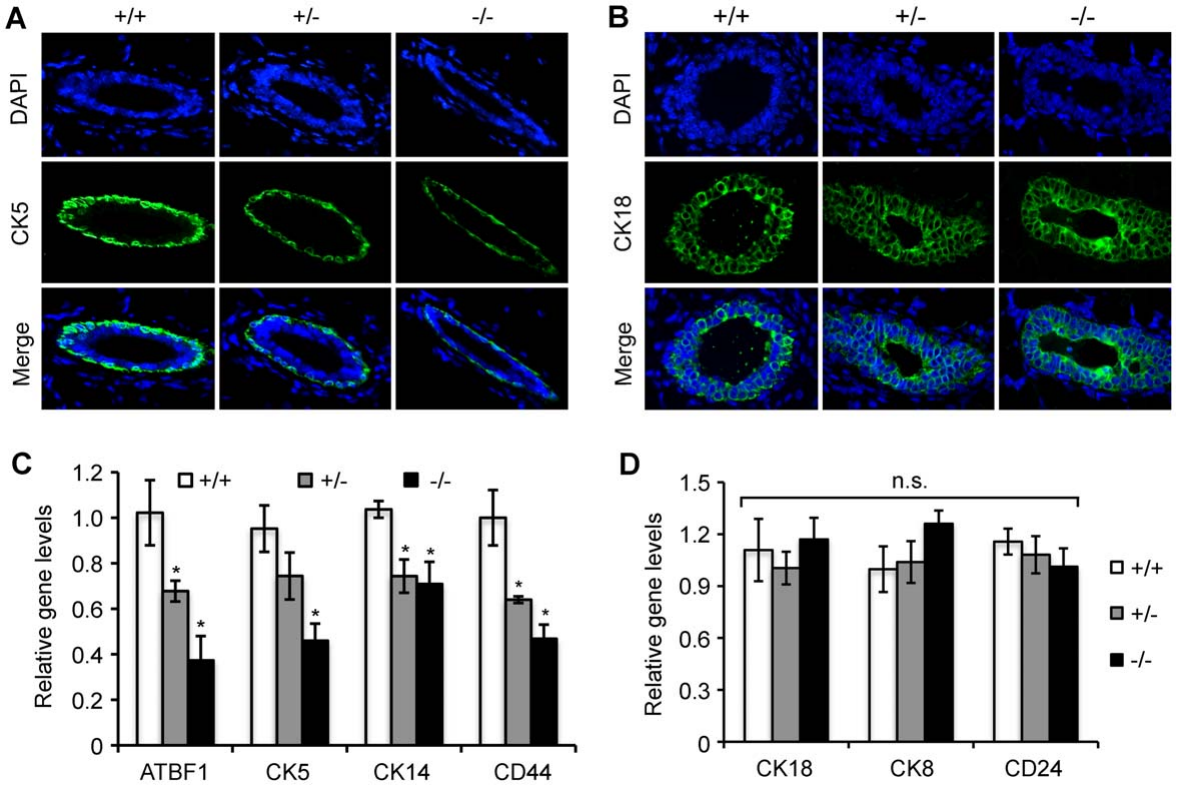
Knockout of *Atbf1* downregulates the expression of basal cell markers in 6-week mammary epithelial cells. (A, B) Detection of CK5 and CK18 by IF staining in mouse mammary tissues. CK5 or CK18 was labeled with FITC (green), and DAPI staining shows nuclei. (C–D) Detection of mRNA levels for basal cell markers *CK5*, *CK14* and *CD44* (C) and luminal cell markers *CK18*, *CK8* and *CD24* (D) by real time RT-PCR in mammary tissues. *Gapdh* was used as the normalization control, and *Atbf1* served as a positive control. Three mice were used for each genotype. *, *P*<0.05 and n.s., *P*>0.05 (one-way ANOVA).

The mammary gland is comprised of an adipose-rich stroma embedded with a branching network of epithelial ducts, which are organized in two distinct cell layers: the basal myoepithelial layer and the luminal epithelial layer. Proper organization of myoepithelial and luminal epithelial cells is crucial for the normal structure and function of mammary glands [35]. Moreover, the myoepithelial layer is not only abundant in stem cells but also associated with cell differentiation during mammary gland development [36], [37], [38] and to suppress tumor development [39], [40]. CK5, CK14 and CD44 as myoepithelial cell markers and CK18, CK8 and CD24 as luminal cell markers are generally used as structural and differentiation markers [41], [42], [43]. Immunofluorescence staining of CK5 and CK18 revealed that knockout of *Atbf1* didn't affect cellular localization of both CK5 and CK18, suggesting that the epithelial organization was not affected by *Atbf1* deletion at this stage. Consistent with results from the *in vitro* model in MCF10A cells, *Atbf1* deletion significantly reduced CK5, CK14 and CD44 expression (Fig. 7A, 7C) but not that of CK18, CK8 and CD24 (Fig. 7B, 7D), as detected by IF staining and real time RT-PCR. These results indicate that Atbf1 regulates the expression of basal cell markers, which may contribute to basal cell maintenance and luminal cell differentiation during the puberty.

## Discussion

### Atbf1 regulates pubertal mammary gland development

Mammary gland development is a highly regulated process involving balanced cell proliferation and differentiation under the control of female reproductive hormones, including estrogen, progesterone and prolactin. During puberty, estrogen is the dominant steroid hormone, and multiple factors have been identified for their effects on mammary gland development. Our findings in this study suggest that Atbf1 is one of the regulators controlling pubertal mammary gland development. First, using an *in vitro* model of mammary epithelial differentiation – the MCF10A cells grown in Matrigel, we found that ATBF1 was induced during cell differentiation, and knockdown of *ATBF1* interfered with the differentiation (Fig. 1, Fig. 2). More directly, *Atbf1* expression increased from week 3 to week 9 during puberty but fell during pregnancy (Fig. 3A), and knockout of *Atbf1* promoted ductal elongation and bifurcation in pubertal mammary glands, which was indicated by extended ductal invasion, total ductal branching, and increased ductal coverage upon the deletion of *Atbf1* in pubertal mammary glands (Fig. 4). During puberty, ductal elongation and bifurcation are accompanied predominantly by cell proliferation. Consistent with the ductal phenotype in the pubertal mammary gland, knockout of *Atbf1* significantly increased cell proliferation (Fig. 5). Taken together, these findings establish Atbf1 as a novel regulator of pubertal mammary gland development.

### The effect of Atbf1 on mammary epithelial proliferation is limited to ER-positive cells

The mammary gland is comprised of ER-positive and ER-negative cells, and estrogen is the most dominant hormone in mammary epithelial proliferation and differentiation during puberty [23]. Several studies have demonstrated that ER-positive cells in normal mammary gland do not proliferate, and estrogen stimulates the proliferation of ER negative cells via a paracrine mechanism [32], [44], which is different from ER-positive breast cancer cells, where ER-positive cells do proliferate. Our findings in this study indicated that knockout of *Atbf1* primarily makes ER-positive cells proliferate (Fig. 6).

The biological function of estrogen is largely mediated by ER, which is primarily associated with ductal elongation rather than lobuloalveolar formation in the mammary gland [45], [46]. Early and complete loss of ER in mammary epithelia prevents the formation of TEBs and severely impairs ductal elongation [47], [48]. While it is not known why and how the estrogen-ER signal stimulates the proliferation of ER-negative but not ER-positive cells, it is possible that Atbf1 could be partially responsible for the lack of proliferation in ER-positive cells because knockout of *Atbf1* increased the proliferation of ER-positive cells (Fig. 6). Our previous studies in which ATBF1 was identified as a functional inhibitor of ER in gene regulation and cell proliferation control and in which an autoregulatory negative feedback loop was established between ATBF1 and the estrogen-ER signaling in ER-positive human breast cancer cells [18], [19], support this prediction. ATBF1 inhibits estrogen-ER signaling via direct interaction with ER, which prevents the binding of the steroid receptor coactivator 3 (SRC3/AIB1) to ER, altering the expression of ER target genes such as CTSD and EBAG9 [20]. In pubertal mammary gland, ablation of *Atbf1* upregulated *Ebag9* (Fig. 6F), but not Ctsd (data not shown).

Knockout of *Atbf1* also significantly upregulated the expression of amphiregulin (*Areg*) (Fig. 6F), an EGF family member transcriptionally induced by estrogen in pubertal mammary glands during the exponential expansion of the ductal system [31]. Areg is a key mediator of the estrogen-driven epithelial cell proliferation and ductal elongation at puberty [31], [49], and it is possible that Areg mediates the acceleration of mammary gland branching and bifurcation upon the deletion of *Atbf1*.

In addition to Areg, two other ER target genes involved in the regulation of cell proliferation, *Igf-1* and *c-Myc* [50], [51], were also upregulated by the deletion of *Atbf1* (Fig. 6F). A number of studies have established the crosstalk between Igf-1 and the estrogen-ER signaling [52], [53], [54]. On one hand, the estrogen-ER signaling stimulates the local synthesis of Igf-1 [50], [55]; while on the other hand, ER transcriptional activity can be induced by the Igf-1 signaling in an estrogen-independent manner [52], [54]. Igf-1 is essential for TEB formation and ductal morphogenesis in pubertal mammary gland [34], [56]. The Myc protein is a well established stimulator of cell proliferation, differentiation and apoptosis [57]. The increase in cell proliferation caused by *Atbf1* ablation could also involve the upregulation of both IGF-1 and Myc. Taken together, these findings support the view that Atbf1 inhibits the proliferation of ER-positive cells by suppressing the estrogen-ER signaling pathway.

Although normal ER-positive cells do not proliferate and estrogen-ER signaling induces the proliferation of ER-negative cells via a paracrine mechanism [44], [58], estrogen-ER signaling is clearly mitogenic and promotes cell proliferation in ER-positive breast cancer cells. It is unknown how this biological change occurs between normal and cancer cells. ATBF1 not only inhibits the proliferation of ER-positive cells in both normal mammary gland and breast cancer cells, it is also frequently inactivated by genomic deletion and downregulation in human breast cancers, and its downregulation is associated with worse patient survival in ER-positive breast cancer [4], [6], [7] (our unpublished data). Therefore, we speculate that molecular machinery exists to limit normal ER-positive cells from proliferating, and ATBF1 is part of this machinery.

### Atbf1 could be more relevant to the differentiation than the proliferation of mammary epithelial cells

In normal mammary gland, expression of ER is an indication of epithelial differentiation, which could be the reason why ER-positive cells do not proliferate. Based on the observation that Atbf1 inhibits ER function [20], increased cell proliferation led by the knockout of *Atbf1* in ER-positive cells could mean a more relevant function of Atbf1 in mammary epithelial differentiation. For example, expression of Atbf1 could cooperate with the estrogen-ER signaling to induce and maintain the differentiation status of mammary epithelial cells. Supporting this idea is the induction of ATBF1 during the differentiation of the MCF10A cells grown in Matrigel, which was accompanied by the expression of differentiation markers (Fig. 1). An increased expression of *Atbf1* in lactating mammary glands (Fig. 3A) could also be related to Atbf1's function in cell differentiation because lactating mammary glands are highly differentiated. A role of ATBF1 in cell differentiation has been suggested by a series of other studies. For example, during brain development Atbf1 expression was increased in the differentiating field when compared to the proliferating stem cells on the ventricular zone [13]. Consistently, when retinoic acid induces neuronal differentiation in the P19 mouse neuroblastoma cell line, Atbf1 expression is elevated by 50-fold within 24 hours [59], [60]. The function of ATBF1 in cell cycle arrest, as demonstrated in other studies [3], [9], could indirectly indicate a role of ATBF1 in cell differentiation *in vivo*.

On the other hand, we found that downregulation of ATBF1 reduced the expression of basal cell markers but not that of luminal cell markers, which was detected in both the human MCF10A cells grown in Matrigel *in vitro* and mouse mammary gland *in vivo*. These results suggest that Atbf1 is needed for the maintenance of basal cells, which has been shown to play a crucial role in cell differentiation. Bocker and his colleagues described a population of normal human breast cells that express cytokeratin-5/6 (CK5/6) and represent stem cells that give rise to both differentiated luminal cells and myoepithelial cells [43]. The adhesion receptor CD44, which is also a stem cell marker, is expressed in the myoepithelium of the developing mammary gland, mediates epithelial-stromal and cell-cell interactions and modulates ductal development [35]. In another study, three distinct human breast epithelial subsets (basal and myoepithelial cells; luminal progenitor cells, and luminal cells) were identified from the resultant lineage-negative (Lin-) population with antibodies against CD49f and epithelial cell adhesion molecule (EpCAM). CK5/6 is expressed in both the basal (CD49f^hi^EpCAM^−^) and luminal progenitor cells (CD49f^+^EpCAM^+^) but not in mature luminal cells (CD49^−^EpCAM^+^), which suggests that CK5/6-positive cells are progenitor cells and may contribute to cell differentiation [61]. In breast cancer cells, myoepithelial cells induce growth arrest and apoptosis with secretion regulation of ECM proteins, anti-angiogenic factors, and protease inhibitors [40], [62]. When co-cultured with breast cancer cells, myoepithelial cells inhibit expression of MMPs [63] and direct polarization and branching morphogenesis during mammary gland development [64], [65].

### Atbf1 could regulate mammary gland development during other stages

The majority of mammary gland development takes place after birth under the control of steroid hormones. Ductal elongation and bifurcation during puberty, which is mostly regulated by estrogen, is only the first major phase. After sexual maturation, recurrent estrous cycles trigger side branching with estrogen and progesterone, pregnancy enhances side branching and induces alveologenesis with lactational differentiation via progesterone and prolactin, and involution occurs at weaning [23], [24]. Our unpublished data suggest that Atbf1 regulates the progesterone-PR pathway and Pg-induced cell differentiation. Moreover, *Atbf1* expression was highly induced during the lactating stage (Fig. 3A). These findings suggest a role of Atbf1 in other stages of mammary development, which remains to be determined.

### Atbf1 could be a novel factor that regulates both mammary gland development and tumorigenesis

Mouse mammary gland development results from balanced cellular activities including proliferation, differentiation, and apoptosis, and disruption of these processes could lead to tumorigenesis. A number of factors have been discovered to have regulatory roles in both mammary gland development and tumorigenesis. For example, the Gata-3 transcription factor, which is specifically located in luminal cells, not only regulates mammary gland morphogenesis and luminal cell differentiation during both puberty and pregnancy [42], [66], it also has been implicated in breast cancer [67]. The oncogenic p120-catenin is crucial for E-cadherin function, and ablation of p120 causes a delay in TEB outgrowth during puberty [68]. Loss of the breast cancer 1 (Brca1) tumor suppressor in mammary epithelium alters the estrogenic growth response, and exposure to increased estrogen or ER activity collaborates with Brca1 deficiency to accelerate ductal elongation and TEB differentiation [69]. Conditional knockout of phosphatase and tensin homolog (Pten) leads to excessive ductal branching and elongation, precocious lobulo-alveolar development, delayed involution, and severely reduced apoptosis as well as neoplasia in mammary glands [70]. Atbf1 has been established as a tumor suppressor by its frequent mutation in human prostate cancer [3] and induction of precancerous lesions upon deletion in the prostate (Sun et al., unpublished data). It has also been implicated in breast cancer by frequent genomic deletion and downregulation [4], [7]. Our findings in this study indicate that Atbf1 is also a regulator of mammary gland development at least during puberty.

In summary, we examined the expression and function of Atbf1 in mouse mammary gland development, and found that Atbf1 was expressed in the nucleus of most mammary epithelial cells with varying levels during different stages of mammary gland development. Knockout of *Atbf1* enhanced ductal elongation and bifurcation, and promoted cell proliferation primarily in ER-positive cells during puberty. In addition, knockout of *Atbf1* significantly reduced the expression of basal cell markers (CK5, CK14 and CD44) *in vitro* and *in vivo*. Taken together, these findings suggest that Atbf1 plays a role in the development of pubertal mammary gland likely by modulating the function of estrogen-ER signaling and the maintenance of basal cells.
